# Association of Pro‐Inflammatory Diets With Increased Risk of Metabolic Dysfunction‐Associated Steatohepatitis: Evidence From a Prospective Cohort Study

**DOI:** 10.1002/fsn3.72109

**Published:** 2026-07-12

**Authors:** Saideh Fathi, Yahya Pasdar, Mehdi Moradinazar, Amir Mehdi Iranshahi, Farid Najafi, Shiva Miryan, Mohamad Sedighi, Narges Shahnazi, Amir Saber, Davood Soleimani

**Affiliations:** ^1^ Nutritional Sciences Department, School of Nutrition Sciences and Food Technology Kermanshah University of Medical Sciences Kermanshah Iran; ^2^ Research Center for Environmental Determinants of Health, Health Institute Kermanshah University of Medical Sciences Kermanshah Iran; ^3^ Faculty of Medicine Medical University of Lodz Lodz Poland

**Keywords:** diet, dietary inflammatory index, inflammation, NAFLD, NASH, steatohepatitis

## Abstract

Chronic inflammation underlies the initiation of steatohepatitis in patients with metabolic dysfunction‐associated steatotic liver disease (MASLD). Diet‐induced inflammation has been highlighted as a contributor to the development of hepatic steatosis. However, its involvement in steatohepatitis risk remains unstudied. This longitudinal analysis included 1130 individuals with MASLD from the Ravansar Non‐Communicable Disease cohort, who were followed from 2015 to 2023. At baseline, MASLD was assessed using the hepatic steatosis index (HSI), a validated non‐invasive scoring system. Usual dietary intakes were collected using a validated food frequency questionnaire (FFQ). The diet's potential to modulate inflammation‐related biomarkers was assessed using the dietary inflammatory index (DII). The acNASH index was employed as a more accurate and effective assessment for steatohepatitis risk in individuals with MASLD. After controlling for confounders, DII scores were significantly associated with changes in weight, ALT, and acNASH scores. Each one‐point increment in the DII score was independently associated with a 0.45% (95% CI: 0.10–0.80; *p*‐value: 0.012) increase in weight, 2.27% (95% CI: 0.19–4.35; *p* = 0.032) increase in ALT, and 1.96% (95% CI: 0.36–3.55; *p* = 0.016) increase in acNASH scores. For each 1% weight gain, the acNASH score increased by 0.63% (95% CI: 0.37–0.88; *p* < 0.001). Weight changes mediated 10.1% of the total relationship between the DII scores and percentage changes in acNASH score (*p* < 0.001). The association between DII scores and acNASH scores remained statistically significant across all strata of gender, socioeconomic status, and physical activity levels (*p* < 0.05). This study demonstrated a direct association between diet‐induced inflammation and increased steatohepatitis risk in patients with MASLD. These findings highlight the importance of promoting anti‐inflammatory dietary patterns and weight management as feasible strategies to reduce the risk of steatohepatitis in patients with MASLD in clinical practice.

## Graphical abstract

The RaNCD longitudinal study showed that adherence to an anti‐inflammatory diet independently predicts significant reductions [Down Arrow] in weight, alanine aminotransferase (ALT), and acNASH scores, indicating improved liver health. However, no significant changes [Double‐sided Arrow] were observed in waist circumference (WC), high‐density lipoprotein (HDL), triglyceride (TG), fasting blood sugar (FBS), diastolic blood pressure (DBP), systolic blood pressure (SBP), or aspartate aminotransferase (AST). Ultimately, these findings highlight the specific hepatoprotective and weight‐loss benefits of adopting anti‐inflammatory dietary patterns.

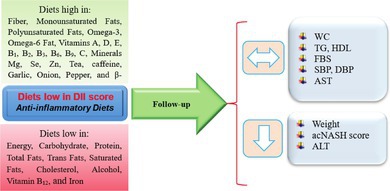

## 1 Introduction

Metabolic dysfunction‐associated steatotic liver disease (MASLD) is identified as the predominant metabolic liver disease. This term was introduced to substitute non‐alcoholic fatty liver disease (NAFLD) to emphasize its connection to metabolic risk factors. It is recognized by abnormal deposition of lipids in hepatocytes, accompanied by one or more metabolic risk determinants (Rinella et al. 2023). A comprehensive review estimated that NAFLD affects nearly 38% of adults worldwide (Younossi et al. 2023). MASLD comprises a spectrum of hepatic histological appearances, from isolated steatosis to complicated steatosis with ballooning degeneration and inflammatory foci (metabolic dysfunction‐associated steatohepatitis, MASH) (Rinella et al. 2023). MASH is a more aggressive state of MASLD and can result in serious health complications such as hepatic fibrosis and cancer. MASH ranks as the second leading reason for liver transplants worldwide, and more than half of liver transplant recipients experience recurrent MASH (Younossi et al. 2021; Younossi et al. 2023). Up‐to‐date clinical guidance emphasizes lifestyle modifications as the foremost treatment approach for patients diagnosed with MASLD and metabolic complications (Arshad et al. 2025). While the first medication has been approved for non‐cirrhotic MASH, lifestyle modifications undeniably enhance the effectiveness of pharmacological therapy (Harrison et al. 2024).

There is substantial evidence that chronic inflammation acts as an essential element in the transition from isolated steatosis to steatohepatitis (Marques et al. 2023). Inflammatory mediators disrupt insulin signaling pathways, contributing to insulin resistance. This disruption decreases glucose uptake by muscles and stimulates lipolysis in adipocytes and de novo lipogenesis from glucose within hepatocytes. Furthermore, inflammation increases oxidative stress, activates immune cells, and triggers lipid peroxidation in the liver (Chen and Zhao 2024; Yu et al. 2016). Therefore, potential therapeutic targets may be found in the modulation of inflammatory pathways in MASLD.

Nutritional evidence supports that nutrients and bioactive compounds in foods can affect health and inflammatory responses (Hossain, Wazed, Asha, et al. 2025; Hossain, Wazed, Shuvo, et al. 2025). The dietary inflammatory index (DII) was designed to measure the net influence of dietary intake on anti‐inflammatory and inflammatory biomarkers. The DII provides a comprehensive assessment of the dietary inflammatory potential, whereas other dietary measures are typically limited to specific nutrients or food groups. This makes the DII particularly suitable for studying diet‐related inflammation in MASLD (Shivappa et al. 2014). The DII has been closely linked to chronic diseases with an inflammatory background, such as diabetes and cardiovascular diseases. A current meta‐analysis reinforces the link between the DII score and simple steatosis (Zhang et al. 2024); however, studies on MASH in individuals with MASLD are still scarce (Li et al. 2023).

Inflammation is a key feature of diet and MASH, but the contribution of diet‐induced inflammation to the transition from simple steatosis to MASH remains unstudied. Knowledge of relations between MASH and the inflammatory capacity of diet can help promote healthier diets in those at risk of MASH. Therefore, this longitudinal cohort aims to evaluate whether the inflammatory capacity of dietary intake is linked to the risk of steatohepatitis among individuals with MASLD.

## 2 Method

### 2.1 Study Design

This longitudinal study analyzed the Ravansar Non‐Communicable Disease (RaNCD) data from 2015 to 2023. The RaNCD cohort, as a component of the national PERSIAN survey, assesses the incidence rates and determinants of non‐communicable diseases (NCDs) in Kurdish individuals. Between 2015 and 2017, the RaNCD cohort recruited 10,047 individuals in midlife (35–65 years) from the Ravansar region (Kermanshah, Iran), based on established selection criteria (Pasdar et al. 2019). Annual monitoring was conducted for participants in the RaNCD cohort to assess the incidence of NCDs. In addition, 3016 participants were randomly subjected to repeated laboratory evaluations during the reassessment phase, following the directive of the PERSIAN committee. We screened these participants based on the inclusion criteria outlined in the multisociety Delphi consensus statement for diagnosing MASLD (Rinella et al. 2023).

The current study received ethical approval from the Kermanshah University of Medical Sciences ethics committee (IR.KUMS.REC.1403.269). Written informed consent was acquired from each participant during enrollment. The RaNCD cohort was executed following the ethical standards outlined in the Helsinki Declaration. We also followed the Strengthening the Reporting of Observational Studies in Epidemiology (STROBE) guidelines to ensure comprehensive and transparent reporting of this study.

### 2.2 Eligibility Criteria

Exclusion criteria in this study were current or previous intake of alcohol, the regular use of corticosteroids, hepatitis B and C viruses, untreated thyroid disease, renal failure, glomerular filtration rate below 60 mL/min/1.7, and unreliable dietary reports (Wu et al. 2021). Furthermore, in accordance with the pre‐specified exclusion criteria, participants with missing values for any of the study variables were excluded from the analysis. Of the 3016 recruited participants, 75 (2.5%) had incomplete data for at least one variable and were excluded.

Participants were also required to have a diagnosis of MASLD, considering the diagnostic threshold of the hepatic steatosis index (HSI) above 36 (Lee et al. 2010). Additionally, participants had to meet at least one cardiometabolic criterion outlined in the multisociety Delphi consensus statement for diagnosing MASLD, including BMI ≥ 25 kg/m^2^ (recommended for Asian populations) or waist circumference WC > 94 cm for men and > 80 cm for women; a SBP ≥ 130 mmHg, DBP > 85 mmHg, or using medications; FBS ≥ 100 mg/dL or using medications; triglyceride ≥ 150 mg/dL, HDL ≤ 40 mg/dL for men and ≤ 50 mg/dL for women, or using medications (Rinella et al. 2023; Tham et al. 2023).

### 2.3 Assessment of Hepatic Steatosis and Steatohepatitis Risk

The NHANES III identified a similar accuracy for non‐invasive diagnostic tests between NAFLD and MASLD (Younossi et al. 2024). In this line, the latest clinical guidance has verified the application of non‐invasive assessments and established cut‐offs from NAFLD to MASLD (Rinella et al. 2023).

We employed the HSI as a validated, non‐invasive, and widely adopted approach for assessing the existence of MASLD in population‐based studies. The HSI score is derived from this equation: 8 × ALT/AST ratio + BMI + 2 (for women) + 2 (for individuals diagnosed with diabetes). A score above 36 indicates the existence of MASLD, with a specificity of 92.4% (Lee et al. 2010).

We also employed the acNASH index as a novel and validated non‐invasive approach for steatohepatitis risk in MASLD (Wu et al. 2021). It is calculated from serum levels of AST and creatinine using the following formula: AST (IU/L) / creatinine (μmol/L) × 10. The acNASH index was originally validated for diagnosing NASH in the Chinese population, but has subsequently undergone external validation across multiple international cohorts (including populations from France, Turkey, Malaysia, Egypt, and Spain), involving 1089 patients of various ethnic backgrounds with biopsy‐confirmed NAFLD. The index maintained robust discriminatory performance across these groups, suggesting that its utility is not restricted to East Asian populations. Furthermore, it has demonstrated greater diagnostic accuracy than other non‐invasive tests, including the HAIR score, the Index of NASH (ION), and the NICE model, in predicting steatohepatitis risk in individuals with NAFLD (Wu et al. 2021).

### 2.4 Assessment of Baseline Dietary Intake and DII Score

Dietary information was obtained from all participants via a validated 118‐item food frequency questionnaire developed for the PERSIAN survey (Eghtesad et al. 2024). The aforementioned FFQ also included an additional section focusing on local food ingredients. Participants reported their average frequency and portion size of each food item over the year prior (e.g., daily, weekly, monthly, or annually) through a face‐to‐face interview. The frequencies of reported consumptions were translated into weights (grams per day) using standard household measures and then converted into energy and nutrient values by the Nutritionist IV software. This study did not include participants with daily energy intake below 800 Kcal or above 4200 Kcal to mitigate the effects of underreporting and overreporting (Banna et al. 2017).

The DII was constructed to investigate the net influence of various diets on inflammation‐related biomarkers (Shivappa et al. 2014). DII score is extracted from articles that examine how different dietary components influence pro‐inflammatory and anti‐inflammatory biomarkers. In total, forty‐five dietary parameters were identified, and each was allocated an “overall inflammatory effect score” based on its capacity to alter inflammation status. Based on FFQ information and the Nutritionist IV software database, only 33 of the 45 original DII parameters were available in this study, including energy, carbohydrate, fiber, protein, fats (total, trans, saturated, monounsaturated, polyunsaturated, omega‐3, omega‐6, and cholesterol), alcohol, vitamins (A, B_1_, B_2_, B_3_, B_6_, B_9_, B_12_, C, D, and E), minerals (Fe, Mg, Se, and Zn), tea, garlic, onion, pepper, β‐carotene, and caffeine. Nonetheless, it has been reported that the exclusion of DII components with marginal relevance to population‐level diets or those not accessible in the Nutritionist IV software database does not significantly confound the connection between DII scores and anti‐ and pro‐inflammatory biomarkers (Ren et al. 2018; Vahid et al. 2017).

In the scoring algorithm of DII, each dietary parameter's intake is initially transformed to a Z‐score. These Z‐scores reflect the deviation of an individual's consumption from the global average, allowing for the determination of the diet's pro‐ or anti‐inflammatory tendency. To neutralize the effect of rightward skew, Z‐scores are recalibrated into percentiles. To achieve a distribution symmetric around 0, the scores are multiplied by 2 and reduced by 1. Each participant's percentile for a dietary parameter is weighted by the corresponding “overall inflammatory effect score”. The calculated values are summed up to yield the final score. Higher DII scores imply a more pro‐inflammatory diet. Diets were classified into anti‐inflammatory (DII < −1), neutral, and pro‐inflammatory (DII > +1) (Petermann‐Rocha et al. 2023).

### 2.5 Demographic, Anthropometric, and Clinical Data

The demographic information was collected by well‐trained interviewers using standardized questionnaires. These variables consisted of gender, age, educational level, urban residence (living within Ravansar city limits), physical activity level (in metabolic equivalents), smoking status, socioeconomic status (SES), medication and dietary supplement intake (including multivitamin minerals, multivitamins, calcium, calcium plus vitamin D, vitamin D, folic acid, omega‐3, iron, and zinc). Anthropometrics were collected from all participants while standing barefoot and wearing minimal clothing. Weight was taken using the bioelectrical impedance analysis device (Inbody 770, Biospace, South Korea). Height was taken with a digital stadiometer (BSM 370, Biospace Co, Seoul, Korea). BMI was calculated by dividing weight by the square of height. WC was taken at the midpoint between the lowest rib and the iliac crest with participants in a relaxed position and gently exhaling, ensuring no compression of the skin or obstruction from clothing. Hip circumference (HC) was assessed at the largest width of the buttocks. Each measurement was made with an accuracy of 0.1 cm. Blood pressure was taken according to the protocol for measuring blood pressure in the PERSIAN Cohort. Participants were instructed to refrain from physical activity, consuming heavy meals, coffee, tea, or alcohol, and smoking for at least 30 min before the measurement. Blood pressure readings were taken after a 10 min rest in a sitting position.

Blood specimens were obtained from individuals after an overnight fasting period. Clotted samples were spun at 3000 RPM for 15 min. The collected serum was then analyzed using an autoanalyzer to measure FBS, lipid profiles, liver enzymes, and creatinine.

### 2.6 Statistical Analysis

The complete‐case analysis was selected to maintain data integrity and analytical consistency. All statistical analyses were performed on the Stata software version 17. Continuous variables were tested for normality using the Kolmogorov–Smirnov test. To determine differences in baseline characteristics among the DII categories, one‐way ANOVA was applied for normally distributed variables, Chi‐squared (χ2) for nominal variables, and Kruskal‐Wallis test for others. Association between DII scores and percentage changes in metabolic risk factors, liver enzymes, and acNASH scores was tested using the generalized linear model (GLM) in both unadjusted and adjusted models. Three adjusted models were defined: *Model I* was controlled for energy intake; *Model II* was additionally controlled for gender; and *Model III* was further controlled for age, follow‐up duration, educational level, SES, smoking status, metabolic equivalents, residence, and medication and supplement use. We also conducted subgroup analyses based on gender (male and female), SES (tertile ranks), and physical activity level (tertile ranks). The structural equation model (SEM) was employed to ascertain the mediating role of weight changes in the relationship between DII scores and acNASH scores. Analyses were conducted using the built‐in SEM command within Stata software. This command is part of Stata's core functionality and was employed to model the relationships between observed and latent variables within the framework of structured linear models. *p*‐values below 0.05 were considered statistically significant.

## 3 Results

**FIGURE 1:**
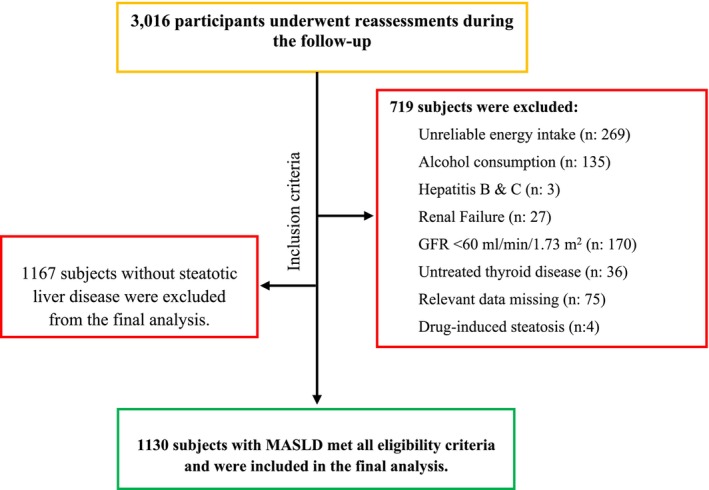
The flowchart diagram of the study.

Figure 1 shows the flow diagram of participant selection. Of the 3016 RaNCD cohort participants subjected to follow‐up biochemical evaluations, 719 individuals did not meet the primary inclusion criteria for study participation. Furthermore, among the remaining participants, 1167 were excluded from the analysis as they were classified as not having hepatic steatosis according to the HSI cut‐off point. A total of 1130 eligible participants with MASLD were considered in this longitudinal analysis. Their mean age (±SD) was 47.51 (±7.85) years at the baseline and 54.55 (±7.81) years at the reassessment. Table 1 shows the baseline profiles of participants according to DII categories. Individuals adhering to an anti‐inflammatory diet (DII < −1) indicated greater weight, BMI, energy intake, and SES ranks than those following a pro‐inflammatory diet (DII > +1). Also, individuals adhering to a pro‐inflammatory diet were more frequently illiterate and more likely to use dietary supplements than those following an anti‐inflammatory diet.

**TABLE 1:** Comparison of baseline characteristics of participants by dietary inflammatory index categories.

Variables	Anti‐inflammatory diet (*N*: 320)	Neutral diet (*N*: 505)	Pro‐inflammatory diet (*N*: 305)	*P*
DII score	1.81 ± 0.56	0.001 ± 0.55	1.72 ± 0.52	0.001
Age; years	46.78 ± 7.84	47.95 ± 8.02	47.56 ± 7.54	0.111
Female; *n*%	249 (77.8%)	391 (77.4%)	249 (81.6%)	0.331
Weight; kg	79.50 ± 12.36	78.61 ± 12.41	75.97 ± 11.94	0.001
Body mass index; kg/m^2^	31.03 ± 3.77	30.74 ± 3.55	30.24 ± 3.69	0.024
Waist circumference; cm	101.99 ± 9.74	103.63 ± 9.15	101.94 ± 9.39	0.013
Hip circumference; cm	104.69 ± 8.52	106.53 ± 7.80	105.53 ± 7.64	0.005
Hepatic steatosis index	40.46 ± 3.67	40.54 ± 3.85	40.31 ± 3.64	0.693
acNASH score	2.53 ± 0.86	2.50 ± 1.05	2.44 ± 0.75	0.445
Physical activity; MET‐h/day	39.94 ± 6.21	39.25 ± 5.96	39.36 ± 4.89	0.222
Energy intake; Kcal/day	2879 ± 635	2432 ± 662	2146 ± 668	0.001
Urban residence; *n*%	287 (89.7%)	365 (72.3%)	151 (49.5%)	0.001^**†**^
Anti‐hyperlipidemic drugs; *n*%	17 (5.3%)	28 (5.5%)	17 (5.6%)	0.987^**†**^
Anti‐hypertensive drugs; *n*%	36 (11.3%)	62 (12.3%)	38 (12.5%)	0.875^**†**^
Anti‐hyperglycemic drugs; *n*%	26 (8.1%)	42 (8.3%)	20 (6.6%)	0.641^**†**^
Dietary supplements; *n*%	76 (23.8%)	131 (25.9%)	105 (34.4%)	0.006^**†**^
Cardiometabolic risk factors				
Overweight/obesity; *n*%	318 (99.4%)	504 (99.8%)	303 (99.3%)	0.537^**†**^
Hyperglycemia; *n*%	72 (22.5%)	142 (28.1%)	66 (21.6%)	0.063^**†**^
Hypertension; *n*%	77 (24.1%)	116 (23%)	80 (26.2%)	0.576^**†**^
Hypertriglyceridemia; *n*%	104 (32.5%)	194 (38.4%)	95 (31.1%)	0.006^**†**^
Low HDL levels; *n*%	203 (63.4%)	304 (60.2%)	185 (60.7%)	0.629^**†**^
Smoking status				0.131^**†**^
Current; *n*%	9 (2.8%)	18 (3.6%)	12 (3.9%)	
Former; *n*%	23 (7.2%)	27 (5.3%)	10 (3.3%)	
Passive; *n*%	117 (36.6%)	203 (40.2%)	140 (45.9%)	
Never; *n*%	171 (53.4%)	257 (50.9%)	143 (46.9%)	
Educational level				0.006^**†**^
Illiterate; *n*%	148 (46.3%)	269 (53.3%)	183 (60%)	
Elementary school; *n*%	34 (28.7%)	141 (27.9%)	83 (27.2%)	
Middle school; *n*%	26 (8.1%)	38 (7.5%)	14 (4.6%)	
High school diploma; n%	34 (10.6%)	34 (6.7%)	14 (4.6%)	
Academic degree; *n*%	20 (6.3%)	23 (4.6%)	6 (2%)	
Socioeconomic status; ranks	2 [2–3]	2 [1–3]	2 [1–2]	0.001^‡^

*Note:* Data are shown as means ± standard deviation, medians [interquartile ranges], or frequencies. *p*‐values were obtained from the one‐way ANOVA test for normally distributed variables. Dietary supplements: Use of vitamin or mineral products ≥ 1 time/week. Residential status: Classified as urban (living within Ravansar city limits) or rural (living in villages/outside city limits) based on national administrative definitions.

Abbreviations: DII, dietary inflammatory index; HDL, high‐density lipoprotein; MET, metabolic equivalent; NASH, non‐alcoholic steatohepatitis.

^†^ Chi‐squared for nominal variables.

^‡^ the Kruskal‐Wallis test.

The mean percentage changes from baseline in metabolic risk factors are presented in Table 2. There was a significant association between percentage changes in weight and DII scores in the unadjusted model. It remained statistically significant across all adjusted models. Each one‐point increment in DII scores was independently linked to a mean increase of 0.45% in weight. Furthermore, a significant association was observed for WC in the unadjusted model. However, this association was no longer statistically significant after controlling for potential confounders. There were no evident associations between DII scores and percentage changes in TG, HDL‐c, FBS, SBP, and DBP in both the unadjusted and adjusted models (*p* > 0.05).

**TABLE 2:** The percentage changes from baseline in metabolic risk factors for each one‐point increment in dietary inflammatory index scores.

Variables		Unadjusted model	Adjusted model 1	Adjusted model 2	Adjusted model 3
Weight; %	β coefficient (95% CI)	0.428 (0.117–0.739)	0.446 (0.109–0.782)	0.521 (0.185–0.857)	0.449 (0.100–0.799)
	*P*	0.007	0.009	0.002	0.012
WC; %	β coefficient (95% CI)	−0.512 (−0.874 – −0.149)	−0.399 (−0.791 – −0.007)	−0.401 (−0.795 – −0.007)	−0.186 (−0.595–0.223)
	*P*	0.006	0.046	0.046	0.372
TG; %	β coefficient (95% CI)	1.45 (−0.788–3.68)	1.81 (−0.612–4.23)	1.97 (−0.467–4.39)	1.96 (−0.607–4.58)
	*P*	0.204	0.143	0.113	0.167
HDL; %	β coefficient (95% CI)	−0.007 (−2.18–2.17)	1.57 (−0.768–3.92)	0.741 (−1.57–3.05)	−1.19 (−1.26–3.65)
	*P*	0.995	0.188	0.529	0.341
FBS; %	β coefficient (95% CI)	0.031 (−1.05–0.992)	0.017 (−1.09–1.13)	−0.069 (−1.18–1.05)	0.186 (−0.998–1.37)
	*P*	0.952	0.976	0.903	0.758
SBP; %	β coefficient (95% CI)	0.066 (−0.562–0.694)	0.073 (−0.607–0.753)	0.088 (−0.596–0.772)	0.029 (−0.699–0.757)
	*P*	0.837	0.833	0.801	0.938
DBP; %	β coefficient (95% CI)	0.178 (−0.502–0.857)	0.229 (−0.506–0.964)	0.358 (−0.377–1.093)	0.106 (−0.668–0.881)
	*P*	0.608	0.541	0.340	0.788

*Note: p*‐values were derived using the generalized linear model. Model 1 was controlled for energy intake. Model 2 was additionally controlled for gender. Model 3 was further controlled for age, follow‐up duration, educational level, socioeconomic status, smoking status, metabolic equivalents, residence, and medication and supplement use.

Abbreviations: CI, confidence interval; DBP, diastolic blood pressure; FBS, fasting blood sugar; HDL, high‐density lipoprotein; SBP, systolic blood pressure; TG, triglycerides; WC, waist circumference.

Table 3 shows the mean percentage changes from baseline in liver enzymes and acNASH scores. There was a significant regression coefficient for percentage changes in serum ALT levels and acNASH scores in the unadjusted model. These associations remained statistically significant across all adjusted models. Each one‐point increment in the DII scores was independently linked to a 2.27% (95% CI: 0.19–4.35; *p* = 0.032) increase in serum ALT levels and a 1.96% (95% CI: 0.36–3.55; *p* = 0.016) increase in acNASH scores.

**TABLE 3:** The percentage changes from baseline in liver enzymes and acNASH scores for each one‐point increment in dietary inflammatory index scores.

Variables		Unadjusted model	Adjusted model I	Adjusted model II	Adjusted model III
ALT; %	β coefficient (95% CI)	3.20 (1.39–5.00)	3.23 (1.28–5.19)	3.43 (1.47–4.40)	2.27 (0.192–4.35)
	*P*	0.001	0.001	0.001	0.032
AST; %	β coefficient (95% CI)	1.41 (−0.120–2.94)	0.948 (−0.704–2.60)	1.16 (−0.496–2.82)	1.54 (−0.209–3.30)
	*P*	0.071	0.261	0.169	0.084
acNASH; %	β coefficient (95% CI)	2.68 (1.29–4.07)	2.55 (1.05–4.06)	2.61 (1.10–4.13)	1.96 (0.359–3.55)
	*P*	0.001	0.001	0.001	0.016

*Note: p*‐values were derived using the generalized linear model. Model I was controlled for energy intake. Model II was additionally controlled for gender. Model III was further controlled for age, follow‐up duration, educational level, socioeconomic status, smoking status, metabolic equivalents, residence, and medication and supplement use.

Abbreviations: ALT, alanine aminotransferase; AST, aspartate aminotransferase; CI, confidence interval; DII, dietary inflammatory index; NASH, non‐alcoholic steatohepatitis.

The relationship between DII scores and percentage changes in acNASH scores according to gender, SES, and physical activity level is shown in Table 4. The association between the DII scores and acNASH scores was significant in both genders. For each one‐point increment in DII scores, acNASH scores were independently increased by 2.61% in males and 0.87% in females. The DII score was linked to percentage changes in acNASH scores in each SES rank. Each one‐point increment in the DII score was independently linked to an increase in acNASH scores of 1.19%, 1.20%, and 0.71% at low, middle, and high SES levels, respectively. The relationship of the DII scores with acNASH scores was significant at each physical activity level. Each one‐point increment in the DII score was independently linked to an increase in acNASH scores of 1.14%, 1.18%, and 1.37% at low, middle, and high physical activity levels, respectively.

**TABLE 4:** Subgroup analyses of the multivariable‐adjusted association between the dietary inflammatory index and percentage changes in acNASH scores.

Variables	Subgroups	*N*	β coefficient (95% CI)	*P*
Gender	Male	241	2.61 (1.58–3.64)	0.001
	Female	889	0.87 (0.63–1.12)	0.001
Physical Activity	Low	371	1.14 (0.74–1.55)	0.001
	Middle	380	1.18 (0.77–1.58)	0.001
	High	379	1.37 (1.01–1.73)	0.001
Socioeconomic Status	Low	378	1.19 (0.79–1.59)	0.001
	Middel	396	1.20 (0.83–1.57)	0.001
	High	356	0.71 (0.27–1.15)	0.002

*Note:* *p*‐values were derived using the generalized linear model. Adjustment for gender, energy intake, age, follow‐up duration, educational level, socioeconomic status, smoking status, metabolic equivalents, residence, and medication and supplement use.

Abbreviations: CI, confidence interval; NASH, non‐alcoholic steatohepatitis.

**FIGURE 2:**
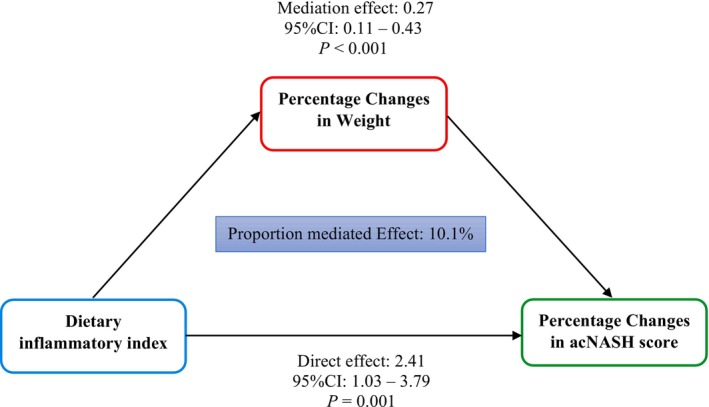
Mediation analysis. The percentage changes in weight mediated approximately 10.1% of the relationship between the dietary inflammatory index and percentage changes in acNASH score.

Figure 2 illustrates the mediating role of weight changes in the association between changes in the acNASH score and the DII score. There was a significant association between percentage changes in weight and acNASH scores. For each 1% weight gain, the acNASH score increased by 0.63% (95% CI: 0.37–0.88; *p* < 0.001). The mediation analysis showed that the percentage changes in weight mediated approximately 10.1% of the total relationship between the DII scores and percentage changes in the acNASH score (*p* < 0.001).

## 4 Discussion

This longitudinal study of the general population revealed a direct association between diet‐induced inflammation and steatohepatitis risk among individuals with MASLD. In this regard, the mean percentage changes in acNASH scores from baseline were +1.96% for every one‐point increment in DII scores. This association remained significant in all strata based on gender, socioeconomic status, and physical activity level and could not be explained by a range of potential confounders and intermediaries such as energy intake. We also observed that a one‐point reduction in DII scores was independently associated with an approximate 0.5% weight loss. Furthermore, weight changes mediated approximately 10% of the total relationship between DII scores and acNASH scores. As far as we are aware, this represents the first research on the role of diet‐induced inflammation in steatohepatitis risk among individuals with MASLD.

There was a significant and independent relationship between inflammatory diets and an increased acNASH score, which is a noninvasive and reliable tool for predicting steatohepatitis in individuals with MASLD (Wu et al. 2021). Meanwhile, we found that each unit reduction in DII scores corresponded to an independent decrease in serum ALT levels of 2.27%. The improvements in the histological features of steatohepatitis are substantially associated with serum ALT levels (Lavine et al. 2011; Sanyal et al. 2010). Many articles have been written on the role of the inflammatory nature of diet in simple steatosis among individuals with MASLD. A literature review revealed that the DII score was positively linked to the risk of developing hepatic steatosis in individuals with MASLD (Zhang et al. 2024). Furthermore, longitudinal data from the RaNCD cohort revealed that inflammatory diets reduced the likelihood of improving hepatic steatosis (Sedighi et al. 2025). Nevertheless, current knowledge about the relation of dietary inflammatory capacity with MASH is scarce and primarily based on evidence from specific nutrients and diets.

Vitamin E, an anti‐inflammatory component of the DII, has been investigated as a potential therapeutic agent for MASH. In the PIVENS trial, vitamin E supplementation (800 IU/day) significantly improved liver enzymes and histological features of steatohepatitis, including steatosis, lobular inflammation, and ballooning (Sanyal et al. 2010). Similarly, the TONIC trial demonstrated reductions in ALT levels, improvement in ballooning, and resolution of MASH in children and adolescents (Lavine et al. 2011); however, no significant improvements in lobular inflammation or steatosis were observed, potentially attributable to age‐related variations in disease severity (Nobili et al. 2016). Importantly, and consistent with our findings, neither trial reported significant improvements in fasting blood sugar or lipid profiles despite the observed histological benefits.

Total fat intake is an inflammatory parameter in the DII scoring algorithm. A case–control study indicated that patients with MASH consumed more fat than healthy individuals (Salehi‐Sahlabadi et al. 2021). Adherence to low‐ and moderate‐fat diets is associated with improvements in the histological features of MASH (Eckard et al. 2013). In contrast, the DII scoring system identifies omega‐3 polyunsaturated fatty acids (PUFAs) as dietary components with significant anti‐inflammatory capacity. The impact of omega‐3 PUFAs on MASH is inconsistent and inconclusive. Some clinical trials have shown that omega‐3 PUFAs are known to have a beneficial effect on hepatic steatosis, ballooning degeneration, and lobular inflammation, as well as inflammatory markers and oxidative stress (Kim et al. 2025; Li et al. 2015), while others have reported no benefits over the control group (Argo et al. 2015; Dasarathy et al. 2015; Sanyal et al. 2014). This controversy may be attributed to variations in the duration, dosage, and composition of omega‐3 PUFAs in supplements, as well as differences in lifestyle modifications within the control groups.

Mediterranean diet (MD) is widely recognized as a health‐promoting eating style with anti‐inflammatory and anti‐oxidative properties. MD has significant potential for preventing and managing chronic conditions such as obesity, diabetes, hyperlipidemia, CVDs, and MASLD (Akhlaghi et al. 2020; Yurtdaş et al. 2022). The foods emphasized in this diet—especially whole grains, legumes, vegetables, fruits, nuts, olives, and seafood—are high in vitamin E, fiber, polyphenol compounds, monounsaturated fatty acids (MUFAs), and omega‐3 PUFAs. A literature review showed that following the MD led to alterations in inflammatory biomarkers (Koelman et al. 2022). Furthermore, an observational study indicated a significant relation between MD scores and the Index of NASH (ION) in individuals with diabetes (Vitale et al. 2022).

Several mechanisms might justify the role of diet‐induced inflammation in MASH. Inflammatory diets, especially those high in fat, particularly trans and SFAs, and low in fiber, can alter the diversity of intestinal flora and impair intestinal epithelial integrity. As a result, the flow of toxic substances such as lipopolysaccharides (LPS) increases into the liver. LPS can stimulate Kupffer cells to produce inflammatory mediators through the Toll‐like receptor 4 pathway (Ren et al. 2024; Yu et al. 2016). These diets can also cause mitochondrial dysfunction and subsequently result in the overproduction of reactive oxygen species (Ofosu‐Boateng et al. 2024). Oxidative stress, through stimulating the c‐Jun N‐terminal Kinases pathway, contributes to hepatocyte apoptosis and fibrogenesis (Hao et al. 2024). In addition, inflammatory diets may trigger NOD‐like receptor protein 3 (NLRP3) inflammasome in Kupffer cells, which leads to exacerbation of inflammation in hepatocytes (Huang et al. 2013). Taken together, diet‐induced inflammation may directly lead to MASH via stimulation of immune responses, induction of oxidative stress, and disruption of gut flora. However, adherence to inflammatory diets may also lead to MASH through an indirect mechanism. Weight management through lifestyle modifications is the main therapeutic approach for patients with MASLD. Weight reduction can independently improve the histological features of MASH (Vilar‐Gomez et al. 2015). We observed that following anti‐inflammatory diets was associated with weight, regardless of possible confounders and intermediaries, including energy intake and physical activity. In line with this finding, a literature review showed that adherence to diets with low DII scores was linked to a lower BMI and obesity risk (Kord Varkaneh et al. 2018). Therefore, part of the relationship between anti‐inflammatory diets and MASH risk in this study could be explained by their influence on weight.

The subgroup analyses (e.g., by gender) yielded interesting findings. Regarding the gender subgroup, our results indicated that the association between DII and acNASH score was stronger in men than in women. Also, in the physical activity subgroup, this association was stronger at the high level than at other levels. Regarding the SES subgroup, the aforementioned association was stronger at the medium status than at the other statuses. Although the exact cause of this phenomenon has not been made clear, it seems to be due to the impact of various factors such as exercise habits, dietary habits, basal metabolic rate, and access to health care services.

The strengths of this longitudinal study include its longitudinal design, well‐defined inclusion criteria, subgroup analysis, and comprehensive control of potential confounding factors. Additionally, the use of the acNASH index further strengthens our study. The acNASH index has demonstrated superior diagnostic performance compared to other non‐invasive methods—including the HAIR score, the ION, and the NICE model—in predicting MASH, based on data from multiple cohorts (Wu et al. 2021). Therefore, this tool enhances the reliability of assessing the risk of MASH in population‐based studies. The study's main limitation is that only 33 of the 45 dietary variables included in the DII score could be obtained from the FFQ and the N4 software. Nonetheless, it has been reported that the exclusion of DII components with marginal relevance to population‐level diets or those not accessible in the N4 program does not significantly confound the connection between DII scores and anti‐ and pro‐inflammatory biomarkers (Ren et al. 2018; Vahid et al. 2017). Additionally, the reliance on the acNASH index rather than liver biopsy represents a methodological constraint. While biopsy remains the gold standard for steatohepatitis diagnosis, its invasiveness, cost, and sampling variability preclude routine use (Wang et al. 2024); thus, the acNASH index should be viewed as a complementary non‐invasive tool rather than a definitive replacement (Wu et al. 2021). Finally, although the longitudinal design is a strength, the follow‐up duration may be insufficient to fully capture the prolonged effects of diet‐induced inflammation on the onset and progression of steatohepatitis in individuals with MASLD.

## 5 Conclusion

This longitudinal study demonstrated a direct relation between inflammatory diets and increased steatohepatitis risk among individuals with MASLD. As a result, promoting the intake of foods that lead to a reduced DII may help decrease the steatohepatitis risk in patients with MASLD. However, long‐term clinical studies are needed to confirm a cause‐and‐effect relationship and to evaluate the effectiveness of such dietary strategies in populations.

## Author Contributions

**Yahya Pasdar:** project administration, data curation, conceptualization, investigation, writing – review and editing, resources, funding acquisition. **Shiva Miryan:** writing – original draft. **Farid Najafi:** project administration, validation, investigation, resources. **Narges Shahnazi:** writing – original draft, investigation. **Saideh Fathi:** writing – original draft, conceptualization, investigation. **Davood Soleimani:** writing – review and editing, conceptualization, investigation, methodology, software, formal analysis, project administration, supervision, visualization. **Amir Saber:** writing – original draft, investigation. **Mehdi Moradinazar:** software, formal analysis, writing – original draft. **Amir Mehdi Iranshahi:** writing – original draft. **Mohamad Sedighi:** writing – original draft, investigation.

## Funding

This research received no financial support.

## Ethics Statement

The current research has been approved by the ethics committee (IR.KUMS.REC.1403.269).

## Conflicts of Interest

The authors declare no conflicts of interest.

## Data Availability

The data that support the findings of this study are available on request from the corresponding author. The data are not publicly available due to privacy or ethical restrictions.
